# Cleansing orthodontic brackets with air-powder polishing: effects on frictional force and degree of debris

**DOI:** 10.1590/2177-6709.21.4.060-065.oar

**Published:** 2016

**Authors:** Brisa dos Santos Leite, Nathalia Carolina Fernandes Fagundes, Mônica Lídia Castro Aragón, Carmen Gilda Barroso Tavares Dias, David Normando

**Affiliations:** 1 Student at the Multi residence in Maxillofacial Surgery and Traumatology Course, Universidade Federal do Rio de Janeiro (UFRJ), Rio de Janeiro, RJ, Brazil.; 2 Masters degree in Dentistry, Universidade Federal do Pará (UFPA), Graduate Program in Dentistry, Belém, PA, Brazil.; 3 Masters degree in Dentistry (Orthodontics), Universidade Federal do Pará (UFPA), Graduate Program in Dentistry, Belém, PA, Brazil.; 4 Assistant professor, Universidade Federal do Pará (UFPA), Department of Mechanical Engineering, Graduate Program in Mechanical Engineering, Belém, PA, Brazil.; 5 Adjunct professor, Universidade Federal do Pará (UFPA), School of Dentistry, Belém, Pará, Brazil. Coordinator, Universidade Federal do Pará (UFPA), Graduate Program in Dentistry, Belém, PA, Brazil.

**Keywords:** Orthodontic bracket, Friction, Dental prophylaxis.

## Abstract

**Introduction:**

Debris buildup on the bracket-wire interface can influence friction. Cleansing brackets with air-powder polishing can affect this process.

**Objective:**

The aim of this study was to evaluate the frictional force and amount of debris remaining on orthodontic brackets subjected to prophylaxis with air-powder polishing.

**Methods:**

Frictional force and debris buildup on the surface of 28 premolar brackets were evaluated after orthodontic treatment. In one hemiarch, each bracket was subjected to air-powder polishing (n = 14) for five seconds, while the contralateral hemiarch (n = 14) served as control. Mechanical friction tests were performed and images of the polished bracket surfaces and control surfaces were examined. Wilcoxon test was applied for comparative analysis between hemiarches at *p* < 0.05.

**Results:**

Brackets that had been cleaned with air-powder polishing showed lower friction (median = 1.27 N) when compared to the control surfaces (median = 4.52 N) (*p* < 0.01). Image analysis showed that the control group exhibited greater debris buildup (median = 2.0) compared with the group that received prophylaxis with air-powder polishing (median = 0.5) (*p* < 0.05).

**Conclusion:**

Cleansing orthodontic brackets with air-powder polishing significantly reduces debris buildup on the bracket surface while decreasing friction levels observed during sliding mechanics.

## RESUMO

**Introdução:**

o acúmulo de detritos na interface braquete-arco pode influenciar no atrito, mas a limpeza dos braquetes usando jatos de bicarbonato de sódio pode interferir nesse processo.

**Objetivo:**

o objetivo desse estudo foi avaliar a força de atrito e a quantidade de detritos remanescentes em braquetes ortodônticos submetidos à profilaxia com jatos de bicarbonato de sódio.

**Material e Métodos:**

avaliou-se, ao final do tratamento ortodôntico, a força de atrito e o acúmulo de detritos na superfície de 28 braquetes de pré-molares. Em uma hemiarcada, os braquetes foram jateados com bicarbonato de sódio (n = 14) por 5 segundos, enquanto a hemiarcada contralateral serviu de controle (n = 14). Foram realizados ensaios mecânicos de atrito e análise imagiológica dos braquetes de ambas as hemiarcadas, a jateada e a controle. O teste de Wilcoxon foi utilizado para a análise comparativa entre as hemiarcadas, com *p* < 0,05.

**Resultados:**

os braquetes submetidos à limpeza com jatos de bicarbonato de sódio apresentaram menor atrito (mediana = 1,27N), quando comparados aos do lado controle (mediana = 4,52N) (*p* < 0,01). A análise imagiológica demonstrou que o grupo controle apresentou maior acúmulo de detritos (mediana = 2,0), em comparação ao grupo que recebeu profilaxia com jatos de bicarbonato (mediana = 0,5) (*p* < 0,05).

**Conclusão:**

a limpeza dos braquetes ortodônticos por meio do jateamento com bicarbonato de sódio reduz significativamente o acúmulo de detritos na superfície deles, bem como os níveis de atrito observados durante a mecânica de deslizamento.

## INTRODUCTION

Fixed orthodontic appliance placement leads to increased biofilm buildup, which hinders oral hygiene practices.^1^ ^,^ ^2^ ^,^ ^3^ As an aid to maintaining patients' oral health, certain prophylactic regimens are performed by professionals,^4^ noteworthy among which is prophylaxis by air-powder polishing (APP).^5^

Since its inception in 1977, prophylaxis by APP has been widely evaluated. This system relies on air, water and sodium bicarbonate to induce proper flow and propel particles to the surface of teeth.^6^ ^,^ ^7^ ^,^ ^8^ Its effectiveness in removing dental plaque and stains has been widely reported in the literature.^9^ ^,^ ^10^

Currently, this technique requires less physical effort, a short clinical period, and does not generate heat compared with rubber cup or Robson brush and prophylactic paste.^5^ ^-^ ^10^ In addition, elastics, archwires and brackets have a decreased risk of breakage.^5^

By removing debris that build up^9^ ^,^ ^10^ on the bracket-wire interface, prophylaxis by APP can influence friction. Friction is a force that slows down or resists the relative motion of two objects in contact with each other. Its direction is tangential to the common boundary of the two surfaces,^11^ and it can reduce or even cancel out tooth movement.^11^ ^-^ ^15^

Debris buildup on the surface of wires^16^ ^,^ ^17^ and orthodontic brackets^18^ increases roughness and produces greater-than-normal frictional force during sliding mechanics. Despite some studies examining the effects of cleaning orthodontic wires,^17^ efficient methods to clean orthodontic brackets and their impact on frictional forces have not yet been investigated. 

Thus, the aim of this study was to assess the effect exerted by cleansing brackets with air-powder polishing (APP) on the levels of debris and frictional forces during sliding mechanics.

## MATERIAL AND METHODS

This study was approved by the Ethics Committee on Research of the Institute of Health Sciences under registration number 039 773/2012. All patients signed an informed consent form before participating. 

Sample size calculation was performed assuming normal distribution of the variables tested. A power of 80% and a bilateral alpha level of 5% was assumed with standard deviation of 0.3 (Group 1) and 0.6 (Group 2). Standard deviation was determined by means of a pilot study with six unused wire segments and six other segments obtained from three patients analyzed after eight weeks of intraoral exposure. Thus, a sample size of 14 bracket-wire pairs (n = 28) per group was deemed adequate.

The effects of blasting brackets with sodium bicarbonate at the end of treatment were evaluated in the 14 bracket-wire pairs. At the end of orthodontic treatment, the finishing wire and brackets (slot 0.022 x 0.028-in) were carefully removed by means of thin cutting pliers (Pin and Ligature Cutter - Standard, Straight / Orthopli Corporation, Philadelphia, Penn, USA).

Before removal of brackets, the hemiarches were randomly divided into two groups: an intervention group with the hemiarch blasted with sodium bicarbonate and a contralateral control group in which the hemiarch was not cleaned. The brackets analyzed were from either Edgewise or Straight-Wire Roth prescriptions (Kirium Line, Abzil, São José dos Campos, SP, Brazil) that varied according to the patient. Aiming to neutralize torque variable in Straight-Wire Roth prescriptions, brackets were passively bonded to the acrylic plates at the friction test performance, which will be discussed later on.

The sample was composed by seven patients randomly selected from an Orthodontics graduate training program in the city of Belém in the Brazilian state of Pará (Brazilian Association of Dentistry, PA, Brazil).

**Figure 1:**
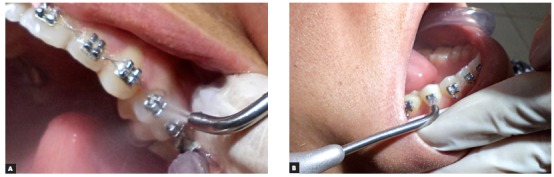
Intraoral view of prophylaxis by air-powder polishing at an angle of 90° relative to the bracket surface in the maxilla (A) and mandible (B).

A handpiece was used for blasting (Practical Jet, Kondortech, São Carlos, SP, Brazil) with bicarbonate composed of sodium hydrogen carbonate, edible colloidal silicic anhydride, and flavoring (Maquira, Maringá, PR, Brazil) for five seconds at a distance of 5 mm and at a 90° angle relative to the bracket surface^5^ (Fig 1). 

The corresponding premolars on both hemiarches were then removed by means of ligature cutting pliers (Pin and Ligature Cutter-Standard, Straight-, Orthopli Corporation, Philadelphia, Penn, USA) across the interface between the bracket base and the adhesive. This technique was tested in a previous study and did not record any significant increase in friction levels.^18^ For confirmation, visual inspections were carried out with the aid of a magnifying glass used to determine the existence of any deformity at the bracket base, which could hinder its correct passive adhesion to the acrylic test plate.

Once the corresponding premolar brackets (n = 28) had been removed, they were carefully bonded individually onto the central area of the extremity of acrylic plates with an area of ​​3 x 5.5 cm and thickness of 0.5 cm. Bonding was performed with adhesive (Adper(tm) Single Bond 2, 3M, Big Lake, Minnesota, USA) and light-curing composite (Orthocem, FGM, Joinville, SC, Brazil) for orthodontic brackets. The brackets were bonded passively, with the slot positioned parallel to the base of the acrylic plate. Subsequently, each bracket was analyzed under a digital optical microscope (MiView MV200UV, Cosview Technologies, Bantian, China) under 120x magnification. 

**Figure 2:**
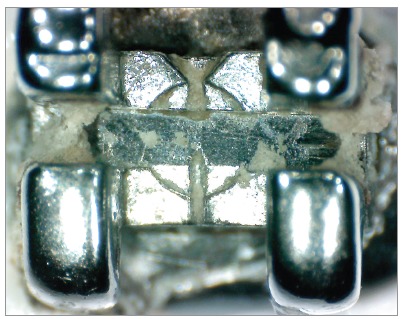
Bracket after clinical use not subjected to prophylaxis by air-powder polishing.

**Figure 3:**
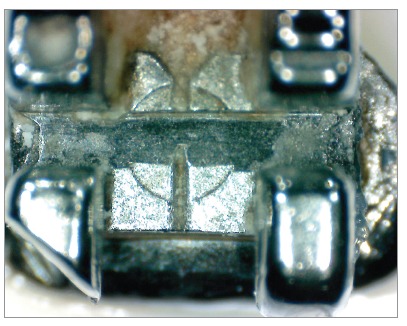
Bracket after clinical use subjected to prophylaxis by air-powder polishing.

The images had scores assigned, according to the presence of debris on the bottom surface of the bracket slots.^16^ A single examiner with expertise in the area assessed the degree of debris. For analysis, we used the following values: 0 = total absence of debris; 1 = some debris involving less than one-fourth of the image analyzed; 2 = moderate presence of debris involving one-fourth to three-fourths of the image; 3 = presence of a large amount of debris involving more than three-fourths of the image examined (Figs 2 and 3).

Thereafter, each wire-bracket pair was subjected to a friction test, carried out by means of a universal testing machine, with a cell capacity of 5 N from 50 N (Emic DL 2000, São José dos Pinhais, PR, Brazil). The friction test was performed with 32 acrylic plates, according to the previously described method.^16^ ^,^ ^17^ ^,^ ^18^ Four conventional as-received brackets corresponding to premolars of the right and left sides (two maxillary and two mandibular) and 28 brackets removed from patients were used. The as-received brackets were bonded individually to four acrylic plates and attached to the grips of the upper specimen holding jaw of the universal testing machine at a fixed point.

**Figure 4:**
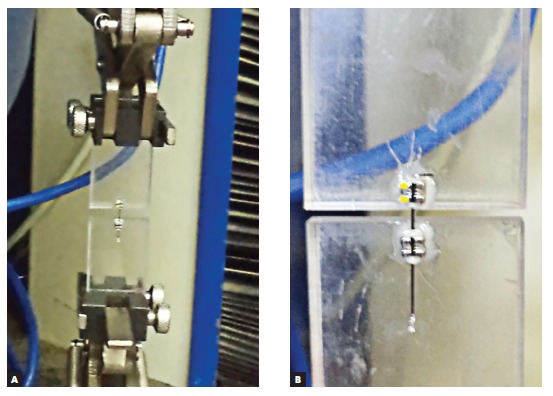
Mechanical friction test being performed: A) upper and lower plates placed at a 90° angle relative to the ground; B) the plate with the as-received bracket was connected at the upper cross-head and the plate with the bracket being tested was attached to the grip at the base.

With each test, the bracket received a new 0.019 x 0.025-in stainless steel wire (Morelli, Sorocaba, SP, Brazil) attached to both brackets by elastic ligatures (diameter 0.120-in, Unicycles(tm), MASEL, Carlsbad, California, USA) and joining the upper and lower plates (Fig 4).

A 5-N load cell was used, with a speed of 0.5 mm/minute for a distance of 10 mm, according to the method described previously.^16^ The kinetic frictional force was measured in Newtons (N) using the mean power. The images and friction tests were performed over a period of 24 hours after removal from the oral environment.

Data were analyzed by BioEstat 5.3 software (Institute for Sustainable Development, Mamirauá, Belém, PA, Brazil). Central tendency and dispersion measurements were obtained for each measure examined after analysis of normal distribution (D'Agostino test), which revealed abnormality in sample distribution (Table 1). Comparison between Group 1 (post-prophylaxis) and Group 2 (control) was performed by means of Wilcoxon test (*p* < 0.05).

**Table 1:** Analysis of normal distribution (D'Agostino test), median, interquartile range (IQR) and *p* value (Wilcoxon test) for kinetic friction force and level of debris of air-powder polishing hemiarch (APP) and control.

Variables	FRICTION			DEBRIS		
	APP	Control	p-value	APP	Control	p-value
n	14	14	< 0.01**	14	14	< 0.05*
Median	1.27	4.52		0.5	2	
IQR	2.71	2.23		2	1	
Normal distribution	< 0.05	< 0.05		--	--	

*p < 0.05; **p < 0.01.

## RESULTS

Analysis of bracket images after orthodontic treatment revealed a statistically significant difference between the surfaces of brackets cleaned with sodium bicarbonate blasting, i.e., air-powder polishing (APP), and the control group surfaces (*p* < 0.05). The median for debris buildup in the control group was 2 (IQR = 1), significantly higher than the medians for the brackets subjected to prophylaxis (median = 0.5, IQR = 2.0) (Table 1). The median for frictional force of the brackets subjected to APP was 1.27 N (IQR = 2.71), significantly lower (*p* < 0.01) than the control group (median = 4.52, IQR = 2.23) (Table 1).

## DISCUSSION

Although orthodontic treatment affords functional and cosmetic correction of teeth, orthodontic brackets, regardless of the material they are composed of, cause accumulation of debris and thus greater-than-normal plaque buildup around them.^1^ ^,^ ^2^ ^,^ ^3^ Professional prophylaxis can be performed as a control method to correct deficiencies in the techniques patients use to brush their teeth.

Debris buildup on orthodontic wires is related to increases in surface roughness and friction.^16^ ^,^ ^17^ Cleaning these wires by rubbing them with steel wool reduces friction.^17^ However, there are few reports regarding the influence of cleaning brackets over friction produced during tooth movement.

APP has proved an effective system for removing debris and stains from surfaces exposed to the intraoral environment.^9^ ^,^ ^10^ *In vitro* studies have been performed to evaluate the effect of sodium bicarbonate particles on surfaces subjected to APP. An increase in friction at the bracket-wire interface was observed after this technique was applied.^19^ ^,^ ^20^ Moreover, debris buildup on the surfaces of orthodontic wires^16^ ^,^ ^17^ or brackets^18^ *in vivo* interferes significantly in friction force produced during sliding mechanics.^16^ ^,^ ^17^

This study examined the degree of friction and amount of debris on brackets after clinical use and prophylaxis by APP, and compared them with brackets that were not subjected to this cleaning system. Visual analysis of debris buildup on the surface of brackets after exposure to the oral environment confirmed the effectiveness of APP. With this technique, bracket slot surfaces exhibited less debris than the group exposed to the oral environment, but which were not cleaned (Table 1, Figs 2 and 3). 

Although friction levels at the bracket-wire interface are directly related to debris buildup on the surface of orthodontic wires,^16^ ^,^ ^17^ previous studies^19^ ^,^ ^20^ that examined as-received brackets revealed changes in the surface of the slots after APP, in addition to increases in surface irregularities and friction. The authors^19^ ^,^ ^20^ reported 15% to 30% increases in friction for metal brackets subjected to APP.

However, friction analysis presents limitations due to the non simulation of a dynamic movement.^16^ ^,^ ^17^ It is important to consider that friction can change during orthodontic movement, and so can the level of debris. An *in vitro* study with an as-received bracket cannot simulate the aging of the material in a clinical situation, and the oral cavity is too complex to be successfully simulated in an *in vitro* test. In this study, the experiment was conducted inside the mouth, and tests were applied into these retrieved brackets. Thus, the results were concluded due to the functional and effective alterations of dental material. The cleansing technique presented more efficiency in the reduction of friction after clinical use, which represents the main evidence in this context. Further studies are necessary to investigate the entire relationship between friction and cleansing methods during clinical use, so as to make appropriate treatment decisions. 

The results of this study showed, however, that cleaning orthodontic brackets with APP produced significant reduction in friction levels. The friction generated by brackets with debris is approximately 355% higher than brackets subjected to cleaning with APP (Table 1). These results show that any increased friction that may be produced by the mechanical action of APP is negligible compared to the reduction in friction that occurs after removal of debris built up on the bracket (Table 1). 

Blasting time, direction, and distance between the device and the bracket were defined based on a previous study.^5^ ^,^ ^6^ Friction produced on the wire-bracket interface showed no significant differences when blasting was applied for 5 or 10 seconds or when it occurred at a distance of 2 mm to 4 mm.^6^ Roughness on the surface of the bracket only becomes visible after 10 seconds of exposure. On the other hand, increased resistance to sliding is directly proportional to exposure time of the surface to sodium bicarbonate blasting, i.e., APP.^19^ Therefore, it seems appropriate to invest five seconds in an attempt to reduce friction caused by the mechanical action of APP. However, our results showed that blasting for five seconds seems insufficient to thoroughly clean brackets, as some specimens still showed considerable debris buildup, even after blasting (Table 1). Nevertheless, the method proposed in this study proved effective in reducing friction during wire-bracket sliding mechanics.

## CONCLUSIONS

Cleansing orthodontic brackets with sodium bicarbonate blasting, i.e., air-powder polishing (APP), for five seconds is an effective method to reduce the levels of debris buildup from the surface of orthodontic brackets as well as to decrease the frictional forces caused by debris observed during and after exposure to the intraoral environment.
